# The Abrasive Wear Resistance of the Segmented Linear Polyurethane Elastomers Based on a Variety of Polyols as Soft Segments

**DOI:** 10.3390/polym9120705

**Published:** 2017-12-12

**Authors:** Konrad Kwiatkowski, Małgorzata Nachman

**Affiliations:** 1 Department of Mechanics and Machine Design Fundamentals, West Pomeranian University of Technology Szczecin, Al. Piastów 19, 70-310 Szczecin, Poland; 2 Institute of Materials Science and Engineering, West Pomeranian University of Technology Szczecin, Al. Piastów 19, 70-310 Szczecin, Poland; Malgorzata.Nachman@zut.edu.pl

**Keywords:** polyurethane elastomers, PUR, abrasive wear, microstructure

## Abstract

The presented results make an original contribution to the development of knowledge on the prediction and/or modeling of the abrasive wear properties of polyurethanes. A series of segmented linear polyurethane elastomers (PUR)—In which the hard segments consist of 4,4′-methylene bis(phenylisocyanate) and 1,4-butanodiol, whilst polyether, polycarbonate, or polyester polyols constitute the soft segments—Were synthesized and characterized. The hardness and wear performance as functions of the variable chemical composition of polyurethane elastomers were evaluated in order to define the relationship between studied factors. The microstructure was characterized in detail, including analysis of the hydrogen bonding by Fourier transformed infrared (FT-IR) spectroscopy and the phase structure by X-ray scattering (WAXS) and differential scanning calorimetry (DSC) methods. The presented studies provide the key features of the polymer composition affecting the abrasive resistance as well as attempts to explain the origin of the differences in the polyurethane elastomers’ performance.

## 1. Introduction

The literature data state quite clearly that, on the basis of the specific mechanical properties of the materials, it is possible to predict the performance and durability of the final product; however, there is an exception—The determination of the wear properties in friction pairs [1,2,3,4,5,6,7]. The huge number of the possible variants of friction pairs and the operating features have not yet allowed the creation of an effective recipe for the prediction of the wear intensity of the friction elements.

The prediction and/or modeling of the wear properties of the synthetic materials, especially polyurethane elastomers (PUR), is particularly difficult, mostly due to the wide variation in their properties [8,9,10,11,12,13]. The use of various raw materials, variable chemical composition, or application of different production methods and further processing methods gives a chance to produce PUR with widely varied properties. In general, the overall mechanical properties of PUR can be quite easily tailored by the thought-out selection of the components, composition, and preparation conditions [14,15,16,17]. For example: The hardness of the final product mainly depends on the content of the hard segments (HS) in the polyurethane. The hardness of the material is higher when the content of the HS is higher. However, in the literature there is a lack of data which could facilitate the prediction and/or modeling of the wear properties of PUR. This extensive research problem is very essential, mainly due to the fact that PUR offer an outstanding abrasive wear resistance when compared with rubbers, plastics, or even metals, and are therefore widely used in the tribological systems where a high abrasion resistance is required. For example: conveyor belt cleaning systems (PU scrapers), vibrating screens (PU sieves), or wheels for heavy duty vehicles, but also in the most severe applications of the aerospace and military industries, and so on [14,16].

Wear and friction are much better understood for metals than they are for polymers. Therefore, researchers try to make direct applications of metal friction and wear to polymers [6,7,10,18]. Applying the results from the wear of metals to polymers suggested that abrasive wear should be related to the hardness [10]. Therefore, the users of PUR are convinced that there is a relationship between the hardness and the abrasion resistance, or, more precisely, if the material has higher hardness, then it is more resistant to abrasive wear. Unfortunately, the literature data do not quite clearly specify this dependency. Zygmunt Wirpsza in his scientific work [14], confirmed the validity of the relationship quoted above.

However, the results obtained in the other abrasion characteristics studies [19,20,21,22] demonstrate a completely different relationship—The abrasive wear resistance of PUR increases along with the content of soft segments (SS), i.e., the elastomers with lower hardness were more resistant to abrasive wear. Nevertheless, the results obtained in our preliminary study [23] showed that the polyurethane elastomers, despite having the same hardness, can differ significantly in their abrasion resistance. It has been shown that the differences in the abrasive wear are substantial (up to 20 times more for materials with the same hardness). The differences in the abrasion resistance are most likely caused by the various components used in the preparation of the final product. This means that the most important factor in the wear resistance of PUR is not their macroscopic hardness but the chemical structure and the resulting morphology.

The segmented chemical structural composition of PUR is the specific structural feature which strongly influences their properties [14,15,16,17]. The molecular structure of PUR is quite complicated. They phase separate during the polymerization reaction, forming a supermolecular structure [24,25,26,27]. Moreover, structural units of various forms and sizes develop during the reaction [28,29,30]. In this study, the microphase-separated structure is analyzed.

The polyurethane chain is composed of the HS and the SS. In most of the polyurethanes, the phase segregation occurs due to the incompatibility (different polarity and chemical nature) between these segments. The SS are composed of polyols which may differ in chemical structure, molecular weight, or functionality. The HS are obtained from isocyanates and low-molecular-weight chain extenders. Practically, only a few isocyanates are used, with 4,4-diphenylmethane diisocyanate (MDI) and toluene diisocyanate (TDI) used most frequently [31]; therefore, it is most likely the chemical structure of the SS that is the most important factor influencing the abrasive wear resistance of the polyurethane elastomers. Although the effect of the chemical structure of the components on the mechanical properties of the polyurethane elastomers has been investigated extensively [32,33,34,35], the effect of the chemical composition and the resulting physical structure on the abrasive wear resistance has not yet been well described [8,9,10,19].

In this study, a range of segmented linear polyurethane elastomers were synthesized with polyether, polycarbonate, and polyester polyols as soft segments. All polyols were characterized by the molecular weight of 2000 g/mol. The HS consisted of 4,4′-methylene bis (phenylisocyanate) (MDI) and 1,4-butanodiol (1,4-BD). The stoichiometric ratio of the isocyanate and hydroxyl groups (NCO/OH ratio) was kept constant. The series of polyurethane elastomers based on the polyether polyol were synthesized with different concentrations of the polyether soft segments in order to assess the relationship between the hardness and the abrasive wear resistance. Additionally, one series of the samples was prepared using a diamine chain extender.

The obtained materials were analyzed for the density, hardness, and abrasive wear resistance. The morphology was characterized by wide-angle X-ray scattering (WAXS), differential scanning calorimetry (DSC), and by Fourier transformed infrared (FT-IR) spectroscopy, the latter being one of the main methods used to describe phase separation in PUR [36,37,38,39,40]. The differences in the chemical structure resulted in the varied physical structure of the polyurethane elastomers and, therefore, in strongly affected wear properties.

## 2. Materials and Experimental Methods

### 2.1. Materials and Synthesis

**Figure 1:**
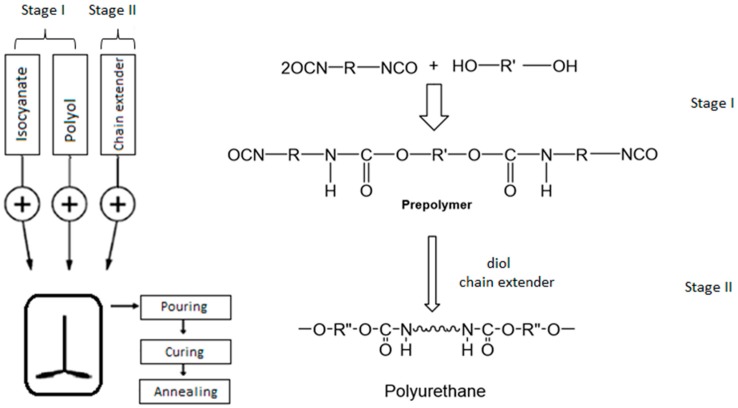
Schematic representation of the synthesis process and the reaction using a two-step polymerization method.

The series of the segmented linear PUR were synthesized using a two-step polymerization method, following the same procedure and conditions as described previously [21]. The first stage of the synthesis involved the preparation of a prepolymer by mixing polyol and isocyanate (with 6 wt % excess) to produce an isocyanate-terminated molecule. In the second stage, a diol or diamine chain extender was added to the prepolymer. Consequently, the polymerization takes place, and produces a multi-block copolymer. A schematic representation of the reaction and the synthesis process using a two-step polymerization method is shown in Figure 1.

As the soft segments, three different linear polyols with various chemical structures were used: the polyether polyol, which was poly(tetramethylene-oxide) (PTMO Terathane^®^ 2000, DuPont, Wilmington, Delaware, USA); the polycarbonate polyol (Desmophen^®^ C 2200, Bayer, Leverkusen, Germany); and the polyester polyol, based on polyethylene glycol and adipic acid (Polios 60/20, Purinova, Bydgoszcz, Poland). All polyols were characterized by the molecular weight of 2000 g/mol. The HS consisted of 4,4′-methylene bis (phenylisocyanate) (MDI, Sigma-Aldrich, St. Louis, Missouri, USA) and 1,4-butanodiol (BD, DuPont, Wilmington, Delaware, USA). A series of poly(ether-urethane)s was synthesized with different concentrations of the polyether SS: 50, 55, 60, 65, 70 wt %. Additionally, one series of the samples was prepared using diamine chain extender—4,4′-methylenebis(2-chlorobenzenamine) (MOCA, Sigma-Aldrich, St. Louis, MO, USA). The molecular structures and detailed specifications of the raw materials are provided in Table 1. The stoichiometric ratio of the isocyanate and hydroxyl groups (NCO/OH ratio) was kept constant—1.05 in all materials. 

The synthesized materials were placed into a mould and left to cure at room temperature for 24 h. Prior to annealing, all samples were heated at 60 ± 5 °C for 2 h in order to complete the reaction. The annealing was conducted for 2 h at the temperature of 100 ± 5 °C. The samples were cooled to room temperature and conditioned for two weeks before the tests. The compositions, the reactants’ molar ratio, and the hardness of the prepared PUR are listed in Table 2.

### 2.2. Methods

#### 2.2.1. The Abrasion Resistance

**Figure 2:**
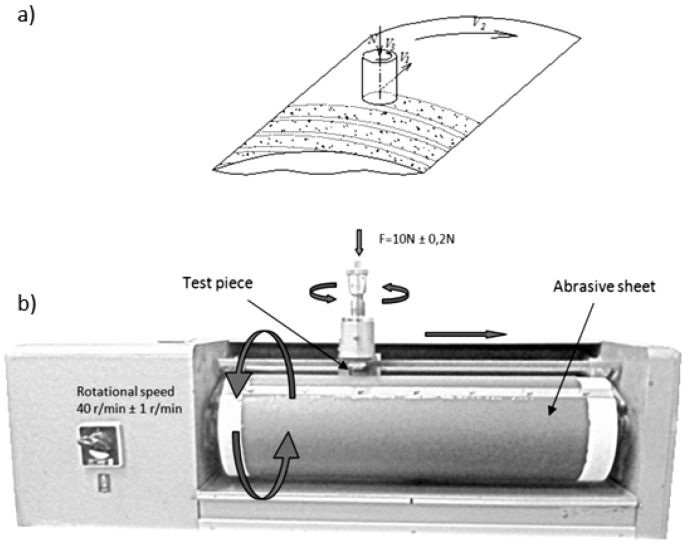
Schematic illustration of (**a**) the test sample sliding over the abrasive sheet and (**b**) the rotating cylindrical roller device.

The abrasion resistance of the test samples against mechanical action upon a surface was assessed by employing a rotating cylindrical roller device (Figure 2) in accordance with ISO 4649:2002 standards. The elastomer test specimen had a cylindrical form, 16 ± 0.2 mm in diameter and 2 mm in height. It was fixed to slide over the abrasive sheet attached to the rotating roller. The sliding distance was 40 m and the sample-abrasive sheet contact pressure was 10 ± 0.2 N. The abrasion resistance was determined as a relative volume loss of the test sample ($\Delta V_{rel}$) compared with the abrasive sheet calibrated using a standard reference. As the standard reference, the rubber compound No. 1 from Federal Institute for Materials Research and Testing (Berlin, Germany) was used.

The abrasive sheet was made of aluminum oxide with a grain size of 60, and it was calibrated to a standard reference compound mass loss of between 180 and 200 mg for an abrasion distance of 40 m. The preparation of the abrasive sheet and its calibration using a standard reference compound was a very important part of the method.

After the abrasion test, the mass loss of the specimen was determined and its volume was calculated from the material’s density. The volume loss of the sample was compared to the results achieved for the reference under the same test conditions. The relative volume loss ($\Delta V_{rel}$) was calculated using the following Equation (1):

(1) $$\Delta V_{rel}=\frac{\Delta m_{t}*\Delta m_{const}}{\Delta m_{r}*\rho_{t}}$$

where $\Delta m_{t}$ is the mass loss of the analyzed sample, mg; $\Delta m_{const}$ is the defined value of the mass loss of the standard rubber sample (defined as 200 mg); $\Delta m_{r}$ is the arithmetic mean of the mass loss of three standard rubber samples, mg; and $\rho_{t}$ is the density of the analyzed material, mg/mm^3^.

#### 2.2.2. The Density

Determination of the density was performed according to ISO 2781:2008.

#### 2.2.3. The Fourier Transform Infrared (FTIR) Spectroscopy

The FT-IR spectra of the urethane elastomers were recorded with a Tensor-27 spectrophotometer (Bruker Optic GmbH, Ettlingen, Germany) equipped with a germanium crystal attenuated total reflectance (ATR) mode. The samples were scanned over the frequency range of 4000–400 cm^−1^ at the resolution of 2 cm^−1^. The carbonyl hydrogen-bonding index (*R*) was determined based on the intensities of the carbonyl stretching vibrations of free (*A_free_*) and hydrogen-bonded (*A_bonded_*) groups located at 1730 and 1700 cm^−1^, respectively. The *R* index was calculated according to Equation (2):

(2) $$R=\frac{A_{bonded}}{A_{free}}$$

the degree of the phase separation (*DPS*) and the degree of the phase mixing (*DPM*) were obtained through Equations (3) and (4).

(3) $$DPS=\frac{R}{R+1}$$

(4) DPM=1−DPS

#### 2.2.4. The Wide-Angle X-ray Scattering (WAXS)

The WAXS, moreover, an investigation was made with the use of wide-angle X-ray diffraction analysis using a diffractometer of the Empyrean (PANalytical, Almelo, The Netherlands) type. Filtered radiation from a lamp of Cu *Kα* and a wavelength of 0.154 nm were used. The step measurement method was used with scattering angles 2*θ* in the range of 10 to 40° and with a step size of 0.1°.

#### 2.2.5. The Differential Scanning Calorimetry (DSC)

The thermal transitions of the investigated polymer materials were studied using the DSC technique (Q100, TA Instruments, Wilmington, DE, USA). The samples were subjected to heating-cooling-heating cycles in the temperature range of −100 to 250 °C. The standard heating rate of 10 °C/min was applied. The melting temperature (*T*_m_) was determined as the maximum of the endothermic peak, while the glass transition temperature (*T*_g_) was set as the midpoint of the heat capacity change.

## 3. Results

The main purpose of this study was to determine the effect of both the chemical composition and microstructure of polyurethane elastomers on their abrasive wear resistance. Understanding of this relationship was crucial to selecting the optimal composition for the most wear-resistant material. In the synthesis of PUR, the aliphatic polyethers or polyesters with molecular weights of 1000–2000 g/mol are mostly used as the SS. In our previous paper [22], we proved that PUR elastomers containing ether segments of *M_n_* = 1800 g/mol revealed the higher abrasion resistance compared with PUR with ether segments of *M_n_* = 1000 g/mol and the same SS content of 60 wt %. For that reason, the materials analyzed in this study contained oligomeric soft segments with the molecular mass of 2000 g/mol. Moreover, 1,4-butanodiol (BD) was used as the chain extender. 

**Figure 3:**
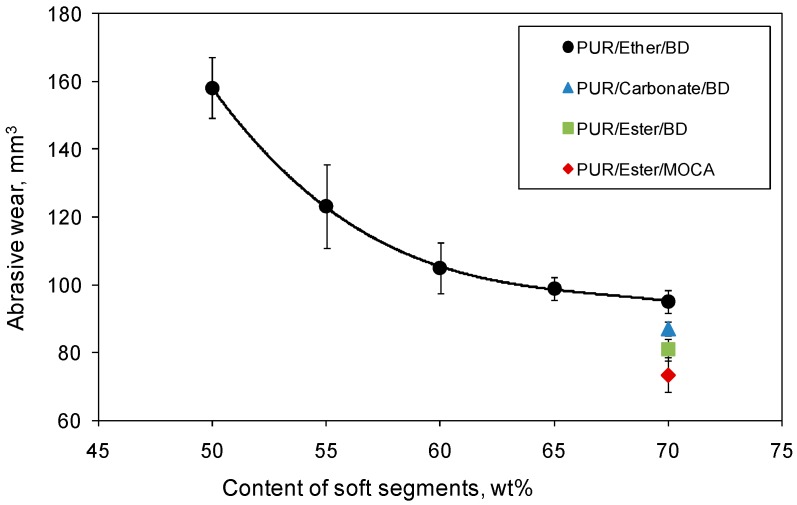
Abrasion wear of polyurethane elastomers (PUR) with various chemical composition and different contents of Soft Segment (SS). The 95% confidence interval is indicated with ISO 2602:1980.

**Figure 4:**
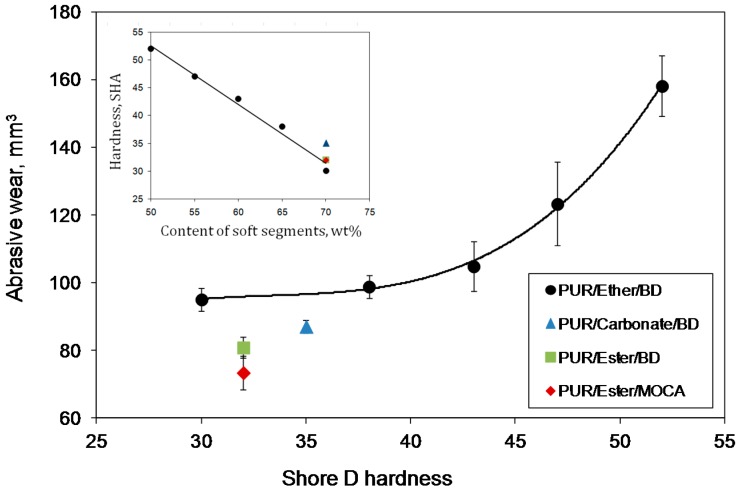
Abrasion wear of PUR versus their hardness.

The results of the wear abrasive resistance were calculated as a sample volume loss after the abrasive test determined from the mass loss and density (Table 3). At first, the effects of the various chemical compositions and different contents of the ether soft segments were studied (Figure 3). When the mass content of SS is increasing within 50–70%, the abrasive wear resistance is also increasing, reaching the highest value at the content of 70%. From these results, we decided that subsequently synthesized PUR materials should contain 70 wt % of SS, and the chemical structure of the polyol was made a variable for further analysis. From Figure 3, it is clear that the polyester soft segments, reaching the value of 81 ± 3 mm^3^, improve the abrasive resistance more effectively than the polyether, for which the value of 95 ± 4 mm^3^ was calculated. Since the ester and carbonate groups reveal a similarity, the PUR elastomer with an aliphatic polycarbonate in the macromolecules was also included in the studies. However, its wear resistance took an intermediate position between polyester- and polyether-containing materials (Figure 4). As the additional modification in the chemical structure of the PUR samples, the diamine chain extender, MOCA, was used instead of BD. The reason for using MOCA was based on the assumption that the resulting PUR would contain the urea groups, which, compared with urethane groups, interact much more strongly with oxygen atoms. The overall results reflected in the abrasive wear resistance of PUR elastomers allowed us to determine that the most suitable composition was based on MDI, Polios 60/20 as the SS with 70% mass content, and MOCA as the chain extender. The average abrasive wear resistance for this sample was 73 ± 5 mm^3^.

The abrasive wear of PUR versus their shore D hardness are presented on Figure 4. For the materials with the same chemical composition (PUR/Ether/BD), the following correlation can be defined: a higher content of the SS results in higher wear resistance. However, when different polyols are used, such a correlation is not observed.

The density of prepared PUR is a superposition of the soft phase density derived from SS, the hard phase density derived from HS, and the interphase density. The densities of the polyols used for synthesis in 23 °C were 0.978 g/cm^3^ for polyether, 1.141 g/cm^3^ for polycarbonate, and 1.187 g/cm^3^ for polyester. In our previous study [22], the density of 1.29 g/cm^3^ for the pure hard phase derived from HS (MDI and 1.4 BD) was determined. Based on this data, it was found that the density values of prepared PUR (Table 3) are consistent with the superposition of all phases and their content in the polymers.

**Figure 5:**
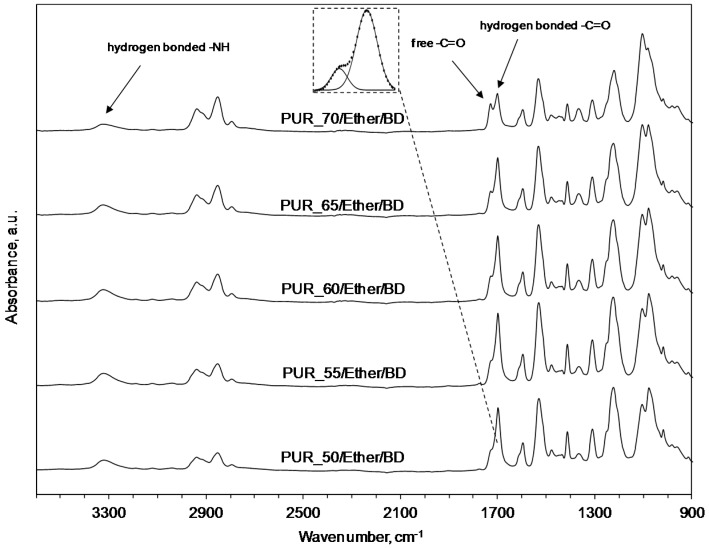
FT-IR spectra of PUR with different content levels of polyether soft segments.

The FTIR spectra for the ether-containing elastomers (Figure 5) indicated that, along with the increase of the SS content, the carbonyl hydrogen bonding index, *R*, is decreasing (Table 4). Specifically, *R* is defined as the ratio of the amount of carbonyl groups connected by the hydrogen bond (1700 cm^−1^) to the amount of free carbonyl groups (1730 cm^−1^). Moreover, while the decrease in the phase separation degree, *DPS*, is observed, the degree of phase mixing, *DPM*, is increasing. This means that, in fact, the urethane hard phase domains, presented in the microstructure, are getting smaller. This also reflects the next correlation: smaller or finer hard phase domains and a higher *DPM* result in higher wear resistance in the case of PUR with polyether SS.

**Figure 6:**
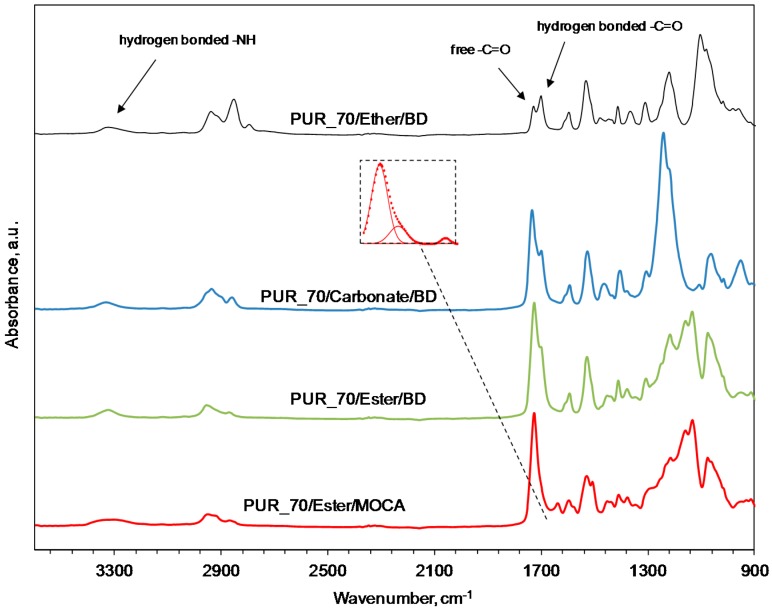
FT-IR spectra of PUR with the various chemical compositions. Contents of the SS for all materials is constant.

When comparing the FTIR spectra of PUR with the content of 70 wt % of different soft segments, presented in Figure 6, substantial differences concerning both the hydrogen-bonded and free carbonyl groups are observed as well as differences in the peak related to the –NH group. If the key factor affecting the abrasive performance of the polymers is the differences in the hydrogen bondings, then it explains our motivation to use the diamine chain extender instead of the butanediol one. Indeed, the amine group, when reacting with the isocyanate, creates the urea group, which is characterized by the presence of –NH groups bonded symmetrically on both sides of the carbonyl group. The two –NH groups belonging to the urea moiety in the HS result in twice as strong hydrogen bonding compared with the one –NH group coming from the urethane group. This is reflected by the widening of the peak corresponding to the wave number in the range of 3300–3330 cm^−1^, and is a consequence of the superposition of two peaks: –NH hydrogen bonding of the urethane group (3330 cm^−1^) and of the urea group (3300 cm^−1^). The peak centered at 1640 cm^−1^ corresponds to the hydrogen-bonded ordered urea carbonyl bond.

**Figure 7:**
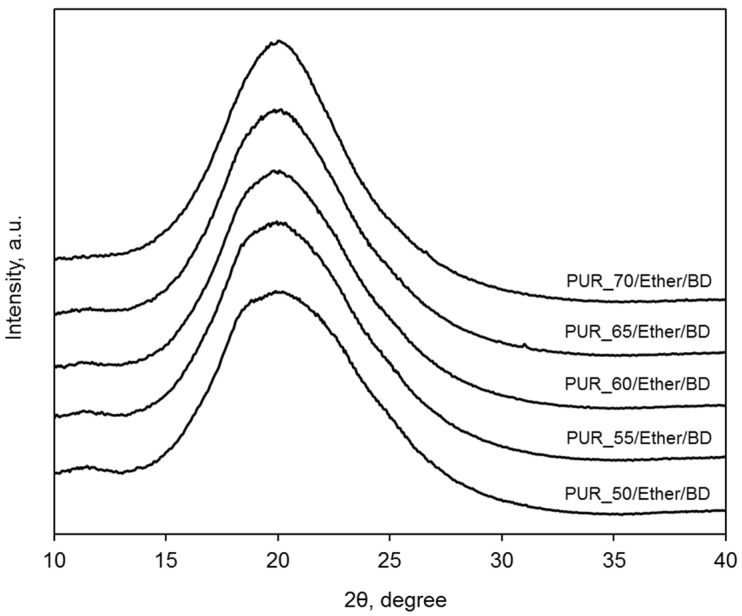
WAXS patterns of PUR with the different contents of the polyether soft segment.

**Figure 8:**
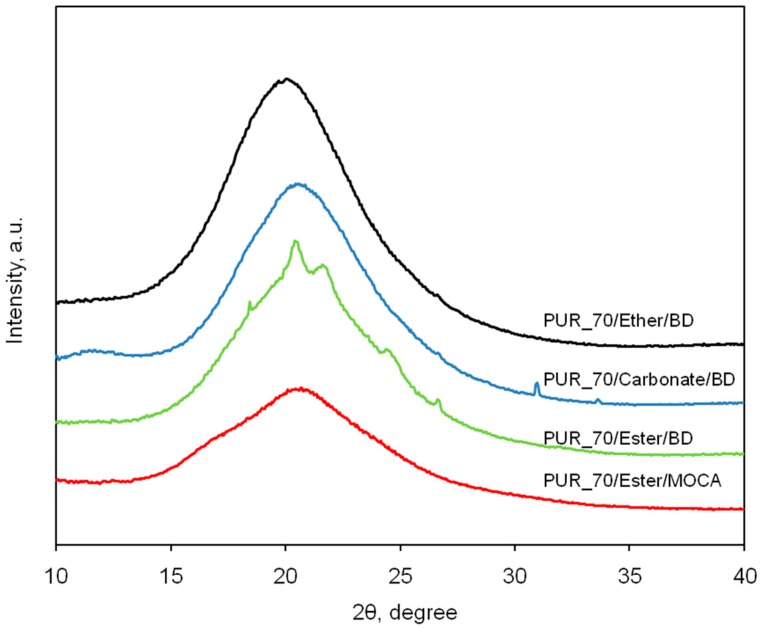
WAXS patterns of PUR with various chemical compositions. Contents of the soft segment for all materials is constant (SS = 70 wt %).

The wide-angle X-ray diffraction profiles (WAXS) of the polyether-containing PUR in Figure 7 do not show a clear effect of the long-range order related to the crystallization of the HS, regardless of the HS to SS ratio. In the case of the elastomer containing 50 wt % of soft segments, the peak on the pattern is slightly broader; this is due to the superposition of two constituent peaks, and might be explained as the effect of the simple (primitive) arrangement. However, it declines along with the content of the soft phase. When we compare the WAXS patterns of PUR samples with different SS presented in Figure 8, it is clear that only the PUR material with the polyester segment and BD as the chain extender reveals the effect of the ordered domains. For the same composition but with MOCA being used, the long-range arrangement is not observed. This is consistent with literature data [10,41,42,43], where MOCA is described as an excellent cross-linker and an outstanding chain extender in polyurethane systems.

**Figure 9:**
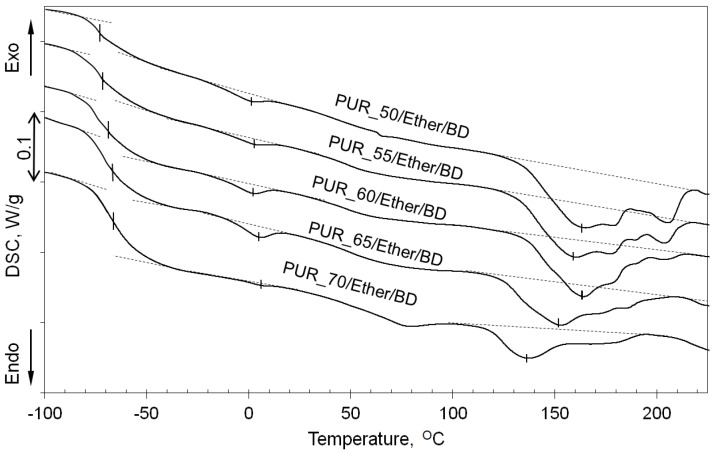
DSC curves of PUR with different contents of the polyether soft segment. First heating.

In order to verify if the crystalline phase in the PUR microstructure is formed by HS or SS, DSC analysis has been employed, and the traces are shown in Figure 9. In the case of the polyether-based elastomers, PTMO segments of *M_n_* = 2000 g/mol make a soft phase exhibiting the glass transition in the temperature range of −73 °C (PUR_50/Ether/BD) to −67 °C (PUR_70/Ether/BD); however, a part of this phase is also crystallized giving small melting endotherms between 0 °C (PUR_50/Ether/BD) and 5 °C (PUR_70/Ether/BD). Obviously, the effect of the glass transition is more pronounced as the soft phase content increases due to more significant changes of the specific heat. Similarly, the lower the content of the HS, the lower are both the melting temperature of the hard phase and the specific heat of this phase transition. Although the ordered domains giving clear WAXS reflections have not been detected for PTMO-based polyurethanes in the whole range of PTMO content, the DSC analysis reveals the evident endothermal effects assigned to the crystals melting during heating.

**Figure 10:**
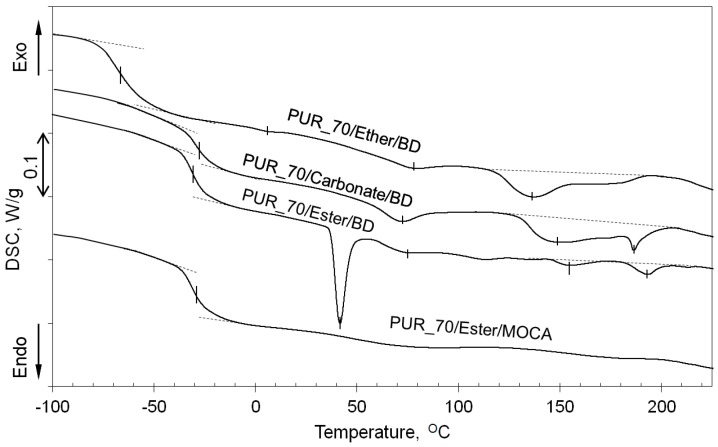
DSC curves of PUR with various chemical composition. Contents of the soft segment for all materials is constant (SS = 70%). First heating.

The DSC thermograms of PUR elastomers with various polyols (Figure 10) indicate the clear melting endotherm of the polyester soft phase when BD is used as the chain extender (PUR_70/Ester/BD). This proves that the diffraction peaks observed on the WAXS profiles are associated with the soft phase. What is interesting is that the same elastomer-containing diamine does not reveal the melting effect of the soft phase, nor the melting of the hard phase which indicates cross-linking of the material. Moreover, it is consistent with the literature data [10,40,41,42] where MOCA is described as an excellent cross-linker.

## 4. Conclusions

In this paper, a range of segmented linear polyurethane elastomers containing polyether, polycarbonate, and polyester polyols as the soft segments were synthesized and characterized. The main purpose was to study the effect of the polyol chemical structure and the content on the abrasive wear resistance of PUR materials in order to explain the origin of their different wear performances. It was confirmed that the higher the content of the soft phase in a PUR sample, the higher the wear resistance. This results in a better phase mixing (higher *DPM* value) in the microstructure. As a consequence, the hard phase domains are smaller or finer and the interfacial area to volume ratio is increasing. These findings became the motivation to continue the studies on PUR elastomers containing 70 wt % of various polyols. The abrasion tests revealed that the polyester soft phase seems to be the most effective in improving the abrasive wear resistance if compared with the polyether and polycarbonate phases. This is explained by the higher cohesion of the polyester phase, because the carbonyl group in ester bonding makes much stronger hydrogen bonds than the ether group. 

It was also concluded that the crystallization ability is not critical for the abrasion wear performance. As the most promising material, we used the polyester-based PUR with the diamine chain extender. The tested sample revealed melting endotherm neither of the soft nor of the hard phase. This is most likely the reason that the calculated degree of phase mixing (*DPM*) is the highest for the selected material. 

## Figures and Tables

**Table 1:** Molecular structure and detailed specifications of the raw materials used for the synthesis.

Raw materials	Molecular structure	Molecular weight, g/mol	Hydroxyl value, mg KOH/g	Melting temperature, °C
Polyether, Terathane^®^ 2000 (PTMO)	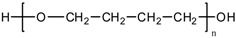	2000	53.4–59.1	28–40
Polycarbonate, Desmophen^®^ C 2200	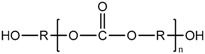	2000	53–59	39–52
Polyester, Polios 60/20	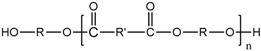	2000	54–58	40–53
Diisocyanate, 4,4′-MDI	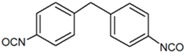	250	-	38–42
1,4-butanodiol, BD	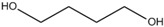	90	-	20.4
Diamine, MOCA	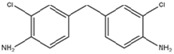	267	-	102–107

**Table 2:** Summary of the prepared polyurethane elastomers’ (PUR) chemical composition and hardness, and the reactants’ molar ratio.

Materials	Composition	Molar ratio: polyol/MDI/chain extender	Soft segment (SS), wt %	Hardness, ShD
PUR_50/Ether/BD	PTMO, BD, MDI	1.00/6.45/5.15	50	52
PUR_55/Ether/BD	PTMO, BD, MDI	1.00/5.33/4.08	55	47
PUR_60/Ether/BD	PTMO, BD, MDI	1.00/4.40/3.19	60	43
PUR_65/Ether/BD	PTMO, BD, MDI	1.00/3.60/2.43	65	38
PUR_70/Ether/BD	PTMO, BD, MDI	1.00/2.93/1.79	70	30
PUR_70/Carbonate/BD	Desmophen 2000, BD, MDI	1.00/2.93/1.79	70	37
PUR_70/Ester/BD	Polios 60/20, BD, MDI	1.00/2.93/1.79	70	32
PUR_70/Ester/MOCA	Polios 60/20, MOCA, MDI	1.00/2.28/1.17	70	32

**Table 3:** The density of prepared PUR. The 95% confidence interval is indicated with SO2602:1980.

Materials	Density, g/cm^3^	95% Confidence interval, g/cm^3^
PUR_50/Ether/BD	1.1274	0.0191
PUR_55/Ether/BD	1.1158	0.0126
PUR_60/Ether/BD	1.1032	0.0070
PUR_65/Ether/BD	1.0912	0.0074
PUR_70/Ether/BD	1.0740	0.0027
PUR_70/Carbonate/BD	1.1535	0.0082
PUR_70/Ester/BD	1.2416	0.0158
PUR_70/Ester/MOCA	1.2508	0.0071

**Table 4:** The carbonyl hydrogen bonding index (*R*), the degree of the phase separation (*DPS*), and the degree of the phase mixing (*DPM*) in the prepared PUR.

Sample	*A*_1730_ *	*A*_1700_ **	*R* ***	*DPS*	*DPM*
PUR_50/Ether/BD	1.40	6.65	4.74	0.83	0.17
PUR_55/Ether/BD	1.73	7.33	4.25	0.81	0.19
PUR_60/Ether/BD	1.70	6.56	3.86	0.79	0.21
PUR_65/Ether/BD	1.57	5.74	3.65	0.79	0.21
PUR_70/Ether/BD	1.79	3.94	2.20	0.69	0.31
PUR_70/Carbonate/BD	10.7	7.29	0.683	0.41	0.59
PUR_70/Ester/BD	13.0	5.62	0.433	0.30	0.70
PUR_70/Ester/MOCA	11.7	3.21 ^+^	0.275	0.22	0.78

*A*: absorption intensity calculated as the area of Gaussian multipeak fitting [absorbance * wavenumber]; * *A*_1730_: absorption intensity of free carbonyl. ** *A*_1700_: absorption intensity of hydrogen-bonded carbonyl. ^+^: the sum of the absorption intensities of hydrogen-bonded carbonyl from ester (1700 cm^−1^) and urea (1640 cm^−1^). *** *R* = *A*_1700_/*A*_1730_: carbonyl hydrogen bonding index.
